# Plasma Proteomic Profiling Identifies Candidate Biomarkers for Pancreatic Ductal Adenocarcinoma

**DOI:** 10.1002/jcla.70313

**Published:** 2026-07-21

**Authors:** Lelang Xiang, Yi Jin, Ran Hu, Yuhang He, Yong Zhang, Ang Li

**Affiliations:** ^1^ Division of Pancreatic Surgery, Department of General Surgery, West China Hospital Sichuan University Chengdu Sichuan China; ^2^ Department of Pancreatic Surgery and Institutes for Systems Genetics, West China Hospital Sichuan University Chengdu Sichuan China

**Keywords:** diagnostic biomarkers, pancreatic ductal adenocarcinoma, plasma biomarkers, proteomics

## Abstract

**Background:**

Pancreatic ductal adenocarcinoma (PDAC) is a highly lethal malignancy that is often diagnosed after curative treatment is no longer feasible. Existing biomarkers, particularly CA19‐9, have limited sensitivity and specificity. Plasma proteins that capture tumor‐associated biological alterations may therefore provide useful signals for earlier detection.

**Methods:**

Plasma samples from 99 patients with PDAC and 30 healthy controls were analyzed using data‐independent acquisition (DIA) proteomics. Differentially expressed proteins were identified using predefined statistical thresholds and further examined by functional enrichment analysis. Selected candidate biomarkers were validated by ELISA in an independent subset.

**Results:**

Among 565 quantified plasma proteins, 52 were differentially expressed between PDAC and controls. These proteins were enriched in extracellular processes, cholesterol metabolism, complement and coagulation cascades, and pancreatic secretion pathways. ELISA validation confirmed higher plasma levels of Cathepsin S, CTRB2, MARCO, PIGR, PRDX6, REG1A, Trypsin‐2, and PEP‐FAP in patients with PDAC compared with healthy controls. ROC analyses showed moderate‐to‐good discriminatory performance for several candidates, and the MARCO + PEP‐FAP model improved classification compared with either marker alone.

**Conclusion:**

These findings reveal circulating proteins linked to key PDAC‐related biological processes and identify eight candidates for further evaluation in multi‐protein diagnostic panels. Larger validation studies incorporating clinically relevant disease control groups are warranted to determine their diagnostic specificity and clinical utility.

## Graphical abstract

Using DIA‐based plasma proteomics followed by ELISA validation, we identified eight circulating proteins that were significantly elevated in patients with pancreatic ductal adenocarcinoma. These proteins reflect key tumor‐associated biological processes, including exocrine dysfunction and immune remodeling, and may serve as candidates for future multi‐marker laboratory assays. Our findings provide a foundation for developing clinically applicable biomarkers to improve PDAC detection.

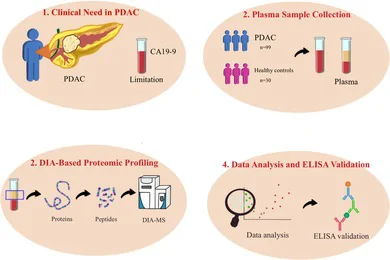

## 1 Introduction

Pancreatic ductal adenocarcinoma (PDAC) remains among the most devastating human malignancies, with a 5‐year survival rate of approximately 13% despite continued progress in systemic therapy and surgical techniques [1]. Curative‐intent resection still offers the most meaningful chance of long‐term survival. Yet, for many patients, this opportunity is lost before diagnosis is even made, as PDAC is frequently detected only after local advancement or distant dissemination [2, 3]. Earlier detection is therefore not merely desirable; it is central to improving resectability and altering the clinical trajectory of this disease [4].

Serum carbohydrate antigen 19‐9 (CA19‐9), although widely used in clinical practice, remains an imperfect biomarker for PDAC [5, 6]. Its diagnostic accuracy is inconsistent, with variable sensitivity and specificity across clinical settings. In addition, CA19‐9 is uninformative in approximately 5%–10% of individuals who are Lewis antigen‐negative [4, 7]. Its interpretation is further complicated by elevations in benign biliary obstruction and inflammatory pancreaticobiliary disorders, conditions that frequently overlap with the early clinical presentation of PDAC [8, 9]. These shortcomings expose a persistent diagnostic gap: the need for circulating biomarkers that more faithfully capture tumor‐associated biological alterations rather than nonspecific downstream consequences.

Proteins are particularly attractive in this context because they represent functional readouts of tumor biology. Alterations in circulating protein abundance may reflect not only malignant transformation and tumor progression, but also host responses, exocrine dysfunction, metabolic rewiring, stromal remodeling, and immune perturbation [10]. With recent advances in high‐resolution proteomic technologies, especially data‐independent acquisition strategies, it is now possible to interrogate the plasma proteome with greater depth, reproducibility, and analytical sensitivity than was previously feasible [11]. Such approaches have accelerated biomarker discovery across multiple cancer types and provide a powerful framework for identifying PDAC‐associated molecular signatures in the circulation [12, 13, 14].

Nevertheless, the translation of plasma protein candidates into clinically useful PDAC biomarkers remains limited. Many reported markers have remained exploratory, with insufficient validation or unclear biological specificity [15]. To address this unmet need, we performed a systematic DIA‐based plasma proteomic analysis, followed by targeted ELISA validation, to identify circulating proteins associated with PDAC. Through this approach, we sought to delineate disease‐related proteomic alterations and nominate candidate biomarkers that may inform the future development of blood‐based diagnostic panels.

## 2 Materials and Methods

### 2.1 Study Population and Sample Collection

This study was approved by the Ethics Committee of West China Hospital, Sichuan University, and was conducted in accordance with the Declaration of Helsinki. Written informed consent was obtained from all participants. Clinical information was retrieved from the electronic medical record system and anonymized before analysis.

Between June 2020 and May 2022, plasma samples were collected from 99 patients with pathologically confirmed pancreatic ductal adenocarcinoma (PDAC) and 30 age‐ and sex‐matched healthy controls. Healthy controls had no history of malignancy, inflammatory disease, or renal dysfunction. For all participants, peripheral blood was drawn after an overnight fast, processed within 2 h, centrifuged to separate plasma, and stored at −80°C until further analysis.

Patients were eligible for inclusion if they had histological confirmation of PDAC from surgical or biopsy specimens and received treatment at the Department of Pancreatic Surgery, West China Hospital. Patients were excluded if they had other malignancies, multiple primary tumors, prior chemotherapy or radiotherapy, use of immunosuppressive or corticosteroid medications, or significant renal disease.

### 2.2 Plasma Processing and Proteomic Preparation

Plasma samples were collected in EDTA tubes and centrifuged at 300 *g* for 10 min at 4°C. The upper plasma layer was transferred to EP tubes and stored at −80°C until further analysis. To reduce batch effects and avoid repeated freeze–thaw cycles, all samples were processed in a single batch immediately after collection.

For proteomic preparation, 2.5 μL of plasma was mixed with 100 μL of ammonium bicarbonate (ABC, pH 8.5) buffer. Proteins were reduced with 2 μL of 1 M DTT at 56°C for 45 min and alkylated with 5 μL of 1 M IAM in the dark at room temperature for 30 min. Samples were then transferred to 30 kDa ultrafiltration tubes, washed with ABC buffer, and digested overnight with trypsin.

The resulting peptides were desalted, dried, and resuspended in 20 μL of Solution A before Data‐Independent Acquisition Mass Spectrometry (DIA‐MS) analysis by Novogene (Beijing, China). Raw data were processed using Spectronaut (v15.0.210615.50606) with a 1% FDR threshold and matched against an in‐house spectral library.

### 2.3 Proteomic Data Analysis

Data preprocessing and statistical analyses were performed using the Wukong omics platform. Missing values were first assessed, and isolated missing values were imputed using the K‐nearest neighbors method. Protein abundance data were then normalized by median normalization.

Exploratory analyses included principal component analysis (PCA) and hierarchical clustering to assess global proteomic variation between groups. Differentially expressed proteins (DEPs) between PDAC patients and healthy controls were identified using *t*‐tests followed by Benjamini–Hochberg correction. Proteins with an adjusted *p* value < 0.01 and a fold change > 1.5 or < 0.67 were considered differentially expressed. Volcano plots and heatmaps were used to visualize the distribution and expression patterns of DEPs.

Functional enrichment analysis of DEPs was conducted using Gene Ontology (GO) biological processes and Kyoto Encyclopedia of Genes and Genomes (KEGG) pathways. GO terms with an adjusted *p* value < 0.01 and KEGG pathways with an adjusted *p* value < 0.20 were considered significant.

To evaluate potential clinical confounding factors, exploratory subgroup analyses were performed within the PDAC cohort. Jaundice status and tumor location showed variable associations with selected protein levels, whereas hypertension, diabetes, smoking, and alcohol consumption were not significantly associated with protein expression patterns (Table S1). Given the possible influence of anatomical tumor location on clinical presentation and circulating biomarker profiles, additional stratified analyses were performed according to tumor site (Table S2). For these analyses, tumors located in the pancreatic head or uncinate process were classified as head‐dominant lesions, whereas tumors arising in the pancreatic neck, body, or tail were classified as non‐head lesions.

### 2.4 ELISA Validation

Candidate proteins were selected from the DIA analysis based on differential abundance (fold change > 2 or < 0.5) and biological relevance. These proteins were evaluated by ELISA in an independent pilot subset of 10 patients with PDAC and 10 age‐matched healthy controls. This subset was used for orthogonal validation to assess whether discovery‐phase candidates showed directionally consistent changes when measured by an alternative assay platform. Given the exploratory nature of this stage, limited sample availability, and assay feasibility considerations, no formal prospective sample size calculation was performed.

Commercially available plasma‐validated sandwich ELISA kits from Shanghai Zoucai Biotechnology Co. and Hefei Bomei Biotechnology Co. were used according to the manufacturers' instructions, and all samples were assayed in duplicate. Standard curves were generated using serially diluted reference standards, and protein concentrations were calculated by linear regression of absorbance values at 450 nm. Group comparisons were performed using independent‐sample *t*‐tests in IBM SPSS Statistics v24.0, with *p* < 0.05 considered statistically significant.

### 2.5 Diagnostic Performance Analysis

Receiver operating characteristic (ROC) curve analysis was performed using the pROC package in R 4.4.2 to evaluate the discriminatory performance of candidate biomarkers. For each marker, the area under the curve (AUC), 95% confidence interval (CI), sensitivity, specificity, and optimal cutoff value were calculated. The optimal cutoff was determined by the Youden index. For consistency, ROC direction was automatically handled by the pROC package when needed.

In the discovery cohort, ROC analyses were performed for the eight proteins subsequently selected for ELISA validation. Exploratory multi‐marker models were then constructed using binary logistic regression to assess whether combined biomarkers improved classification performance. MARCO and PEP‐FAP were selected for combined analysis because they showed favorable individual discrimination and represented biologically distinct PDAC‐related processes: MARCO reflecting immune and macrophage‐associated remodeling, and PEP‐FAP reflecting extracellular matrix remodeling and stromal proteolytic activity. Their potentially complementary sensitivity and specificity profiles further supported evaluation as a combined diagnostic model. Where available, CA19‐9 was also incorporated into comparative ROC analyses to improve clinical interpretability.

In the independent ELISA subset, additional exploratory ROC analyses were conducted to assess whether the discriminatory trends of candidate biomarkers remained directionally consistent when measured by an orthogonal assay platform. Given the limited sample size of this subset, these analyses were considered supportive and hypothesis‐generating rather than definitive estimates of diagnostic performance.

## 3 Results

### 3.1 Basic Characteristics of the Included Population

A total of 129 participants were included, comprising 99 patients with PDAC and 30 healthy controls. Age, sex, and BMI were comparable between the two groups. Among patients with PDAC, the median CA19‐9 level was 415.4 U/mL (range, 0.5–4981.0 U/mL), and 28.3% presented with clinical jaundice.

Tumors were located in the pancreatic head in 45% of patients, the uncinate process in 11%, the neck in 8%, and the body or tail in 35%. For exploratory subgroup analyses, tumors in the pancreatic head and uncinate process were grouped as head‐dominant lesions, whereas tumors in the pancreatic neck, body, or tail were grouped as non‐head lesions. Detailed baseline characteristics are presented in Table 1.

**TABLE 1:** Baseline characteristics of patients with PDAC and healthy controls.

Variable	Category	PDAC (*n* = 99)	Healthy controls (*n* = 30)	*p*
Age, years		62.39 ± 11.00	60.90 ± 6.20	0.350
BMI, kg/m^2^		24.01 ± 2.40	23.34 ± 2.30	0.154
Sex	Male	58 (58.6)	17 (56.7)	0.853
	Female	41 (41.4)	13 (43.3)	
Comorbidities	Hypertension	22 (22.2)	7 (23.3)	0.899
	Diabetes mellitus	19 (19.2)	4 (13.3)	0.467
Lifestyle factors	Smoking history	33 (33.3)	8 (26.7)	0.496
	Alcohol consumption	27 (27.3)	6 (20.0)	0.428
Tumor size, cm	≤ 2	12 (12.1)	—	
	> 2 to ≤ 4	57 (57.6)	—	
	> 4	30 (30.3)	—	
CA19‐9, U/mL	≤ 30	19 (19.2)	30 (100.0)	< 0.001
	> 30 to ≤ 300	44 (44.4)	0 (0.0)	
	> 300 to ≤ 1000	33 (33.3)	0 (0.0)	
	> 1000	3 (3.0)	0 (0.0)	
Clinical jaundice	No	71 (71.7)	30 (100.0)	< 0.001
	Yes	28 (28.3)	0 (0.0)	
Tumor location	Head	45 (45.5)	—	
	Uncinate process	11 (11.1)	—	
	Neck	8 (8.1)	—	
	Body/tail	35 (35.4)	—	

*Note:* Data are presented as mean ± SD or *n* (%), as appropriate. *p* values were calculated using independent‐samples *t*‐tests, chi‐square tests, or Fisher exact tests, as appropriate. Variables not applicable to healthy controls are indicated by em dashes.

Abbreviations: BMI, body mass index; CA19‐9, carbohydrate antigen 19‐9; HC, healthy control; PDAC, pancreatic ductal adenocarcinoma.

To examine potential clinical influences on circulating protein levels, exploratory subgroup analyses were performed within the PDAC cohort. PIGR, Cath‐S, and MARCO were significantly higher in jaundiced than in non‐jaundiced patients, whereas PEP‐FAP, PRDX6, CTRB2, REG1A, Trypsin‐2, and CA19‐9 did not differ significantly according to jaundice status (Table S1). In the tumor‐location analysis, CTRB2, PIGR, and Cath‐S differed significantly between head‐dominant and body/tail tumors, while PRDX6, MARCO, and Trypsin‐2 showed no significant location‐related differences (Table S2).

### 3.2 Proteomic Profiling and Identification of Differentially Expressed Proteins

**FIGURE 1:**
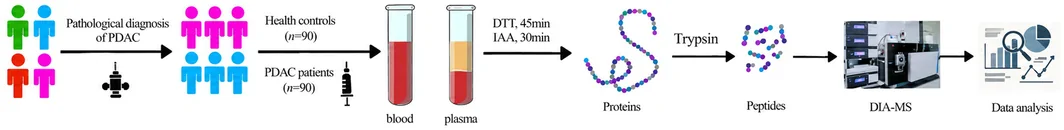
Overview of the plasma proteomics workflow for PDAC biomarker discovery. Plasma samples from patients with pancreatic ductal adenocarcinoma (PDAC; *n* = 99) and healthy controls (*n* = 30) were analyzed using data‐independent acquisition mass spectrometry (DIA‐MS). Differentially expressed proteins were identified by statistical screening and further assessed by functional enrichment analysis. Selected candidate proteins were subsequently validated in an independent subset using enzyme‐linked immunosorbent assay (ELISA).

**FIGURE 2:**
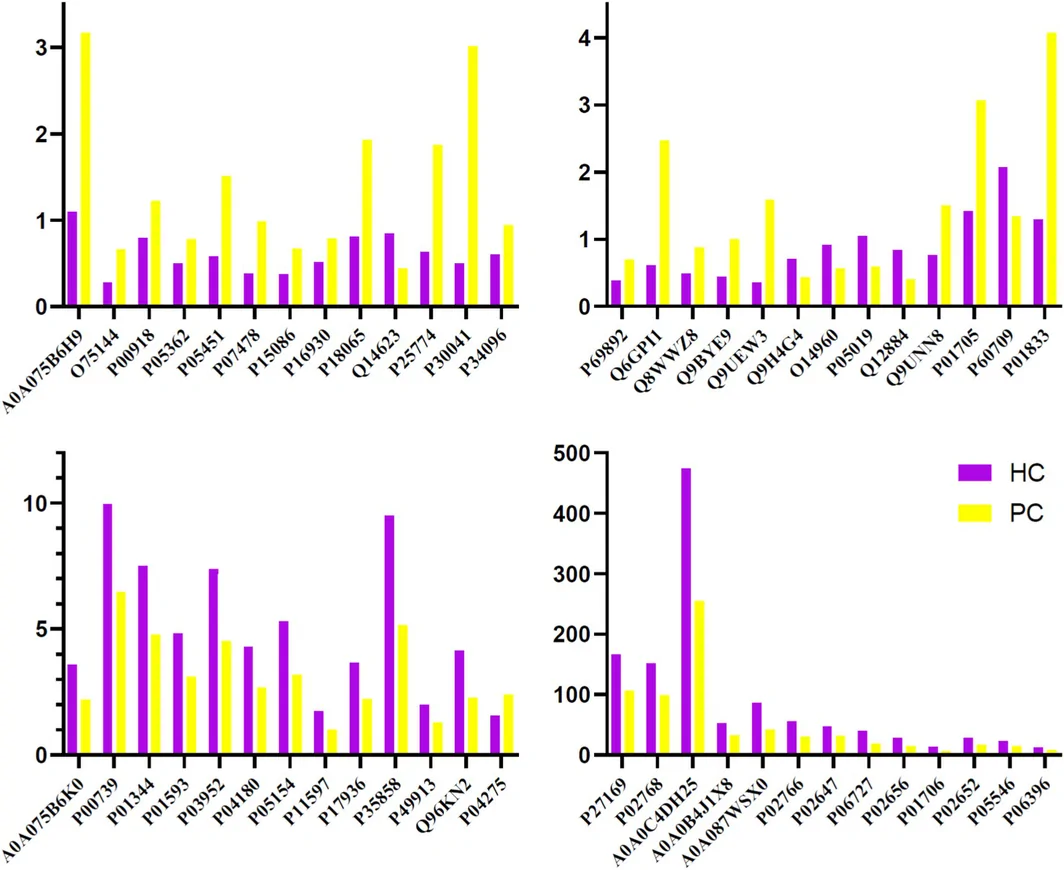
Differential protein expression patterns between PDAC and healthy controls. Differentially expressed proteins (DEPs) were identified between patients with PDAC (*n* = 99) and healthy controls (*n* = 30). Proteins meeting the predefined criteria of adjusted *p* < 0.01 and fold change > 1.5 or < 0.67 were classified as significantly upregulated or downregulated.

DIA‐based proteomic profiling identified 565 plasma proteins across all samples (Figure 1). Using an adjusted *p* value < 0.01 and a fold‐change threshold of > 1.5 or < 0.67, 52 proteins were identified as differentially expressed between patients with PDAC and healthy controls, including 21 upregulated and 31 downregulated proteins (Figure 2).

**FIGURE 3:**
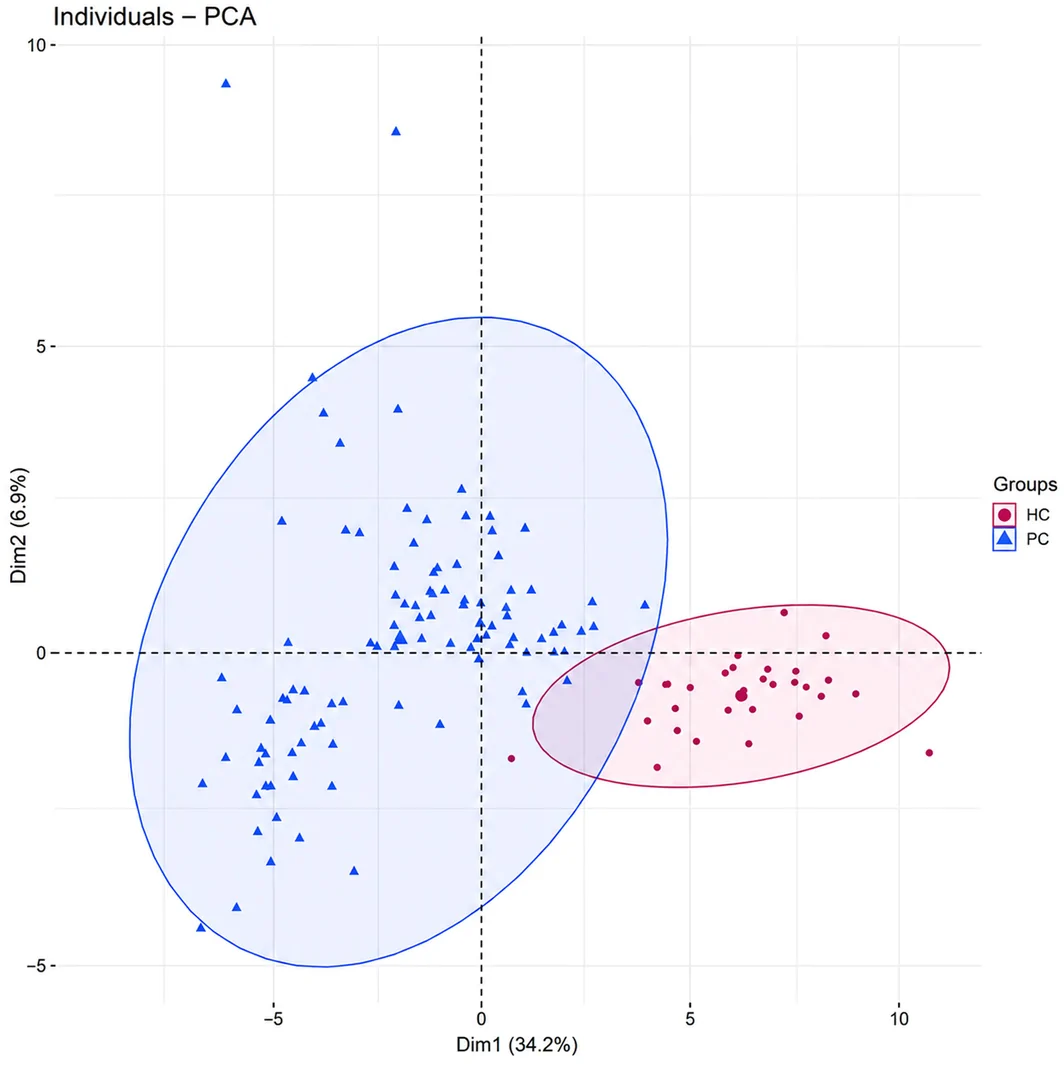
Principal component analysis of differentially expressed plasma proteins. Principal component analysis based on 52 differentially expressed proteins showed clear separation between PDAC and healthy control samples, indicating distinct plasma proteomic signatures. Each dot represents an individual plasma sample.

**FIGURE 4:**
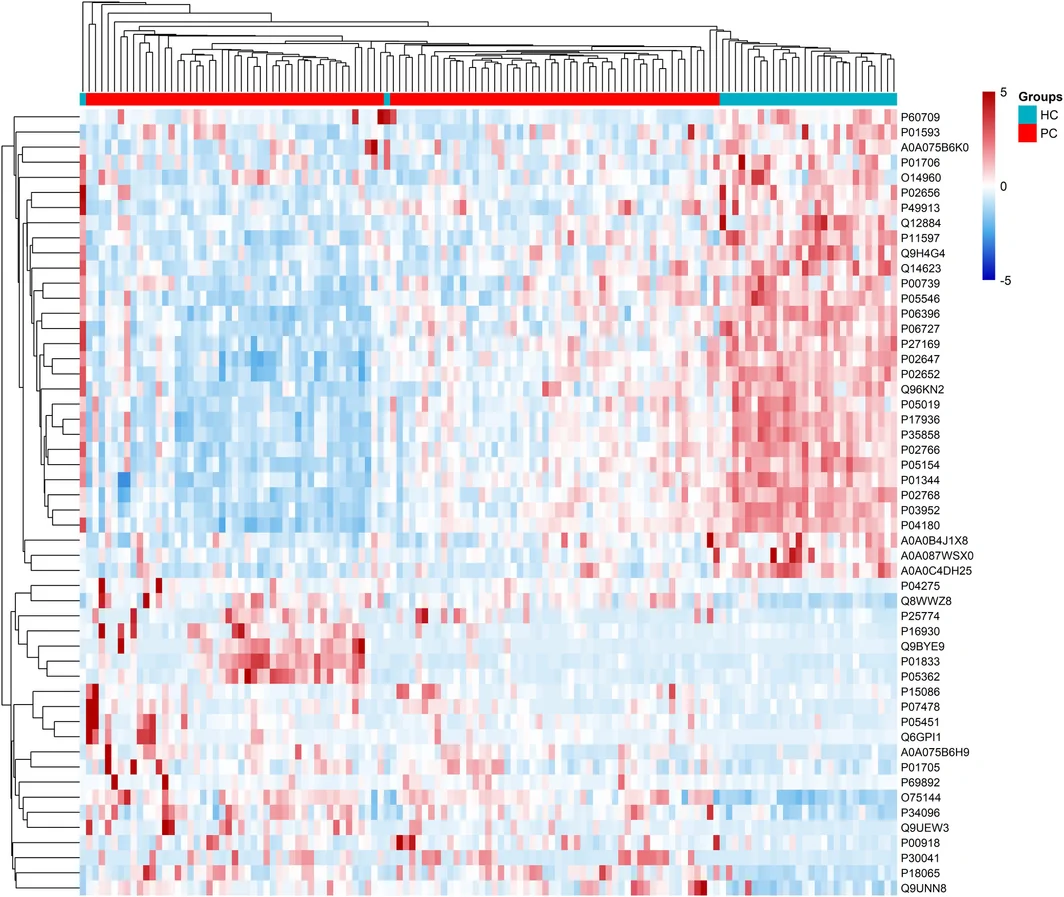
Hierarchical clustering heatmap of differentially expressed plasma proteins. Heatmap showing the expression profiles of 52 differentially expressed proteins across PDAC and healthy control samples. Distinct clustering patterns indicate group‐level separation and consistent expression differences. Color gradients represent relative protein abundance.

**FIGURE 5:**
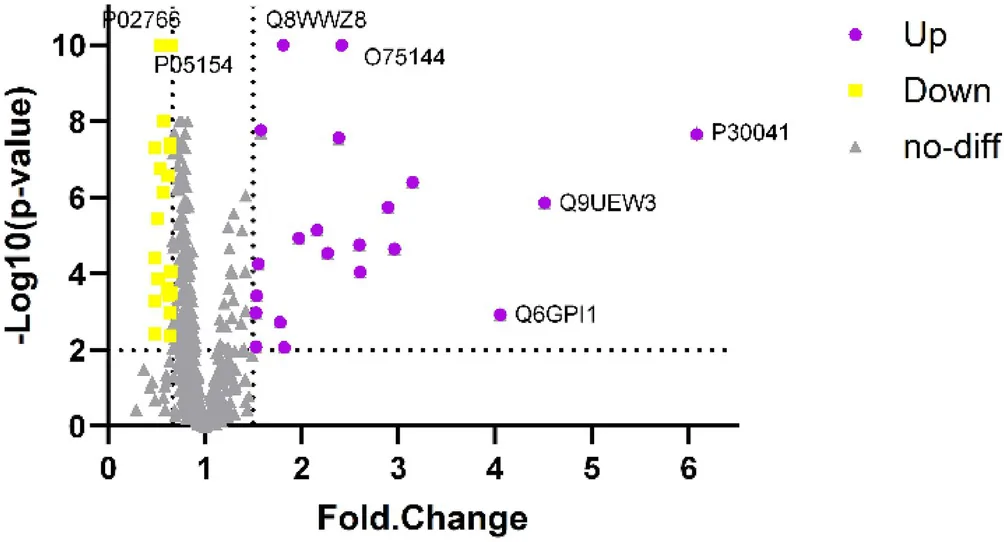
Volcano plot of plasma protein expression in PDAC versus healthy controls. Volcano plot showing the distribution of 565 quantified plasma proteins. Upregulated proteins in PDAC are shown on the right, and downregulated proteins are shown on the left. Proteins with adjusted *p* < 0.01 and fold change > 1.5 or < 0.67 were considered significantly altered.

Based on these 52 differentially expressed proteins, principal component analysis showed clear separation between PDAC and control samples (Figure 3), suggesting distinct disease‐associated plasma proteomic profiles. Hierarchical clustering yielded a similar pattern, with samples from each group displaying relatively consistent expression signatures (Figure 4). The volcano plot further illustrated the distribution of all quantified proteins and highlighted the differentially expressed proteins that exceeded the predefined statistical and fold‐change thresholds (Figure 5).

### 3.3 Functional Enrichment Analysis

**FIGURE 6:**
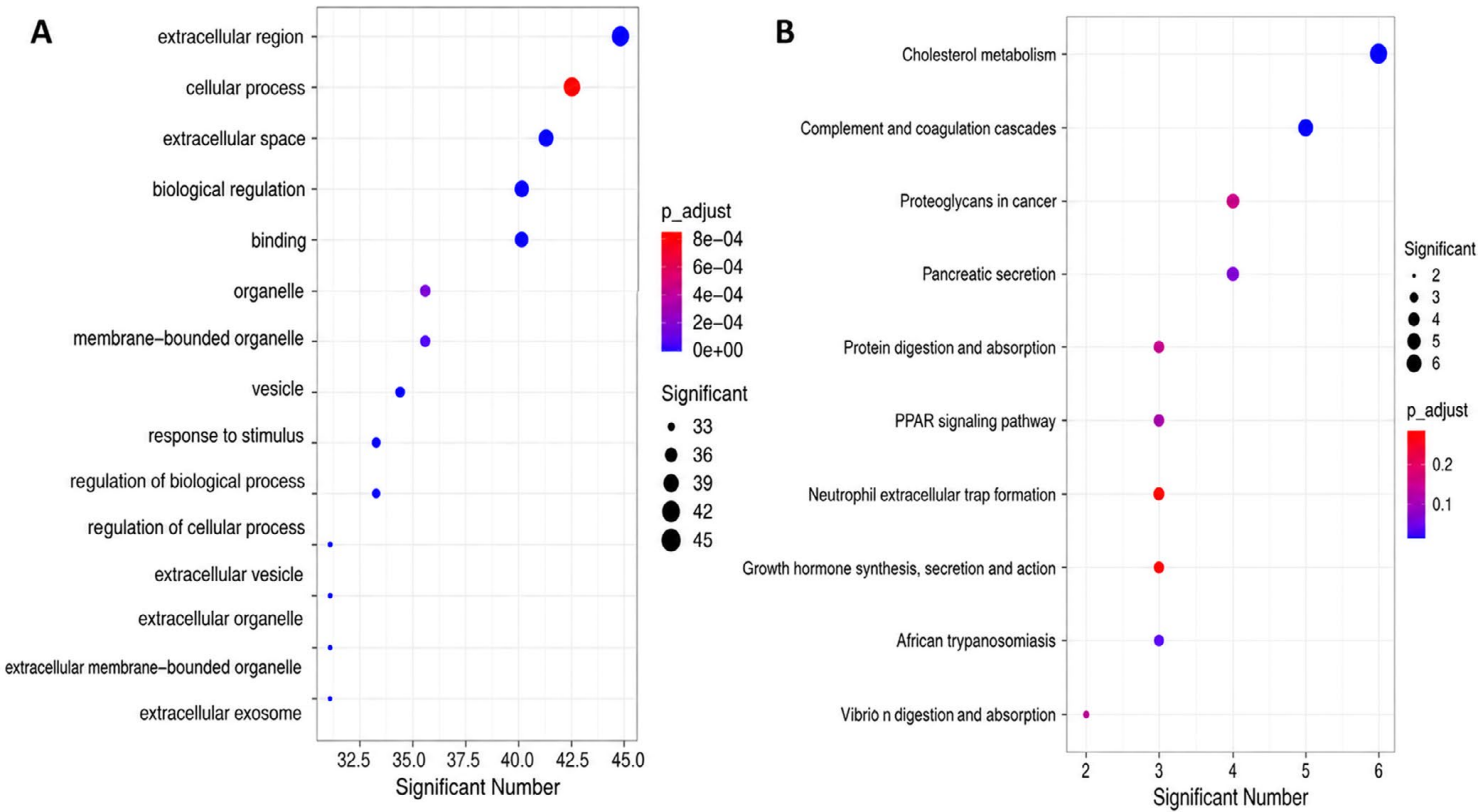
Functional enrichment analysis of differentially expressed proteins. (A) Gene Ontology (GO) biological process enrichment analysis showed significant involvement of extracellular regions, vesicle‐related processes, and immune‐associated functions (adjusted *p* < 0.01). (B) Kyoto Encyclopedia of Genes and Genomes (KEGG) pathway analysis revealed enrichment in cholesterol metabolism, complement and coagulation cascades, and pancreatic secretion pathways (adjusted *p* < 0.20).

Functional enrichment analysis showed that the differentially expressed proteins were mainly involved in extracellular and vesicle‐related biological processes. KEGG pathway analysis further identified significant enrichment in cholesterol metabolism, complement and coagulation cascades, and pancreatic secretion pathways (Figure 6). Together, these pathway‐level alterations suggest that the circulating proteomic profile of PDAC reflects coordinated disturbances in metabolic regulation, inflammatory activation, and exocrine pancreatic function.

### 3.4 ELISA Validation of Candidate Biomarkers

Proteins showing marked differential abundance in the DIA analysis, together with biological relevance, were selected for ELISA validation in an independent subset of 10 patients with PDAC and 10 matched controls. Seven proteins—cathepsin S (Cath‐S), chymotrypsinogen B2 (CTRB2), macrophage receptor with collagenous structure (MARCO), polymeric immunoglobulin receptor (PIGR), peroxiredoxin‐6 (PRDX6), lithostathine‐1‐alpha (REG1A), and trypsin‐2—were significantly elevated in PDAC plasma. Prolyl endopeptidase fibrinolytic activity protein (PEP‐FAP) was also significantly elevated in PDAC plasma (*p* < 0.01; Table 2).

**TABLE 2:** Plasma concentrations of candidate proteins measured by ELISA.

Biomarker	Healthy controls (*n* = 10)	PDAC (*n* = 10)
APOA4, μg/mL	19.083 ± 5.602	38.217 ± 18.552*
Cathepsin S (Cath‐S), mmol/L	65.589 ± 19.272	128.424 ± 59.173**
CTRB2, ng/mL	3.958 ± 1.370	7.739 ± 3.341**
IGFBP2, ng/mL	66.243 ± 23.373	140.508 ± 72.939*
MARCO, ng/mL	0.722 ± 0.248	1.680 ± 0.709**
PIGR, ng/mL	1.900 ± 0.607	3.954 ± 1.142**
PRDX6, ng/mL	1.273 ± 0.323	2.064 ± 0.452**
REG1A, ng/mL	72.904 ± 18.980	126.755 ± 49.206**
Trypsin‐2, ng/mL	27.134 ± 8.452	55.241 ± 14.912**
CDHR2, μg/mL	7.347 ± 2.121	5.572 ± 1.414*
PEP‐FAP, pg/mL	1086.099 ± 229.082	1594.044 ± 454.643**
ICOSL, pg/mL	261.980 ± 82.613	222.803 ± 127.858

*Note:* Values are presented as mean ± SD. Group comparisons were performed using independent‐samples *t*‐tests.

Abbreviations: ELISA, enzyme‐linked immunosorbent assay; HC, healthy control; PDAC, pancreatic ductal adenocarcinoma; SD, standard deviation.

*p < 0.05 and **p < 0.01 versus healthy controls.

ApoA4 and IGFBP2 showed moderate increases (*p* < 0.05), whereas inducible CO‐stimulator ligand (ICOSL) did not differ significantly between groups. Overall, ELISA confirmed the directionally consistent differential expression of eight key proteomic candidates, including eight significantly elevated proteins. Given the pilot nature of this independent subset, these findings should be regarded as orthogonal supportive validation rather than definitive clinical confirmation.

### 3.5 Diagnostic Performance of Candidate Biomarkers in the Discovery Cohort

ROC analyses were performed in the discovery cohort to further evaluate the discriminatory performance of the validated candidate biomarkers. Several proteins showed moderate‐to‐good diagnostic ability. Among individual markers, MARCO achieved a relatively high AUC, while trypsin‐2, REG1A, and PEP‐FAP also demonstrated favorable discrimination. CA19‐9 showed the highest individual diagnostic performance among the evaluated markers.

**FIGURE 7:**
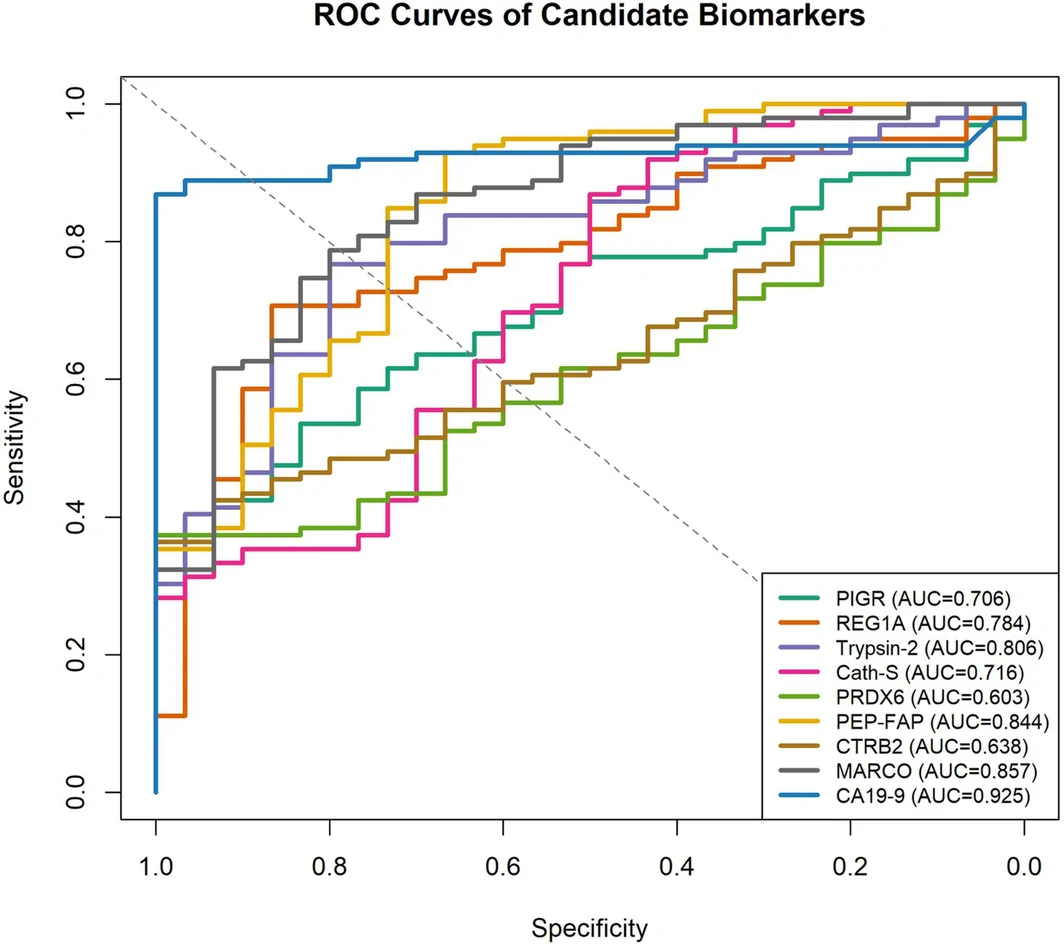
Receiver operating characteristic curves of candidate plasma biomarkers in the discovery cohort. Receiver operating characteristic (ROC) curves were generated for candidate plasma biomarkers in the discovery cohort, including 99 patients with PDAC and 30 healthy controls. CA19‐9 showed the highest area under the curve (AUC = 0.925), followed by MARCO (AUC = 0.857), PEP‐FAP (AUC = 0.844), and Trypsin‐2 (AUC = 0.806). Additional AUC values were 0.784 for REG1A, 0.716 for cathepsin S (Cath‐S), 0.706 for PIGR, 0.638 for CTRB2, and 0.603 for PRDX6. The diagonal dashed line indicates no‐discrimination performance (AUC = 0.50).

**FIGURE 8:**
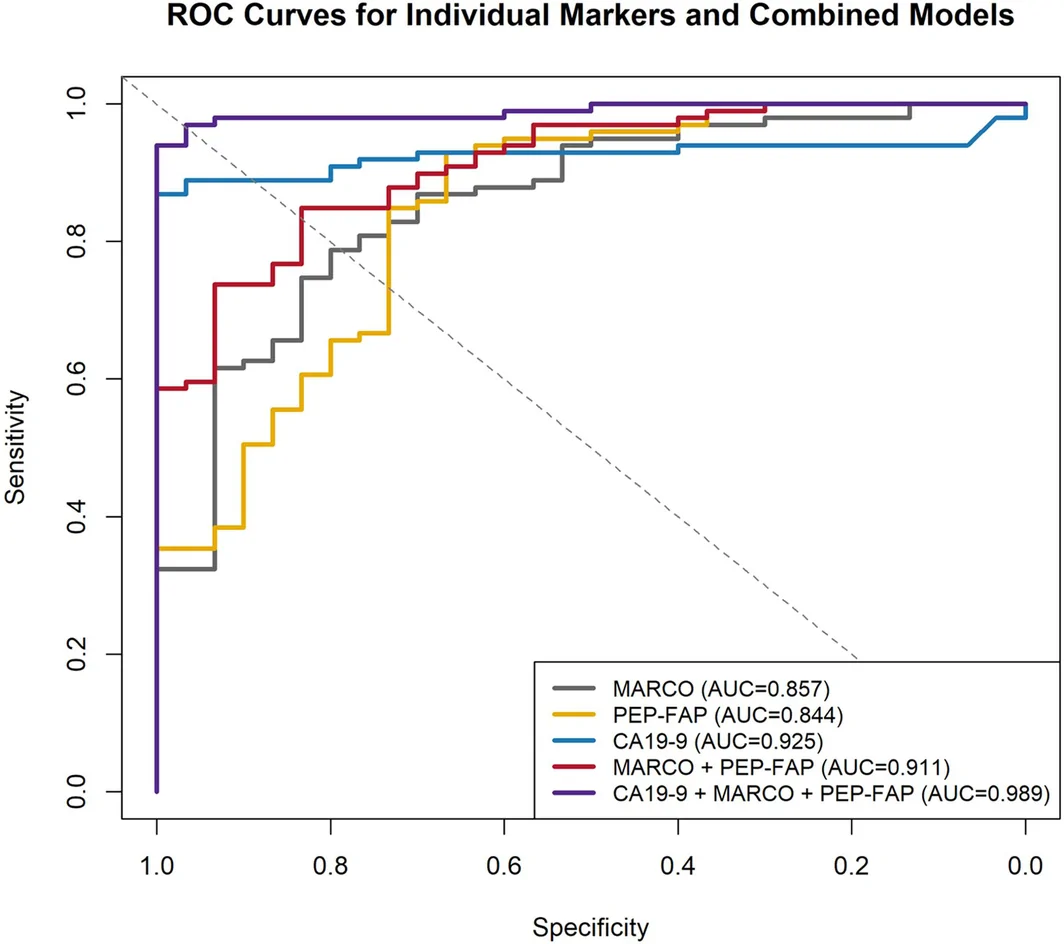
Receiver operating characteristic curves of selected individual biomarkers and combined diagnostic models in the discovery cohort. ROC curves were generated for selected individual biomarkers and combined diagnostic models in the discovery cohort, including 99 patients with PDAC and 30 healthy controls. Among individual markers, CA19‐9 showed the highest discriminatory performance (AUC = 0.925), followed by MARCO (AUC = 0.857) and PEP‐FAP (AUC = 0.844). The combined MARCO + PEP‐FAP model improved classification performance (AUC = 0.911) compared with either marker alone, and the addition of CA19‐9 yielded the highest overall performance (AUC = 0.989). The diagonal dashed line indicates no‐discrimination performance (AUC = 0.50).

To determine whether biologically complementary markers could improve classification, exploratory multi‐marker models were constructed using logistic regression. MARCO and PEP‐FAP were selected for combined analysis because they showed favorable individual AUCs and represented distinct PDAC‐related biological processes: MARCO reflecting immune and macrophage‐associated remodeling, and PEP‐FAP reflecting extracellular matrix remodeling and stromal proteolytic activity. The MARCO + PEP‐FAP model achieved a higher AUC than either marker alone, supporting the value of integrating immune‐related and stromal remodeling–related signals within a single diagnostic model. Adding CA19‐9 further improved the AUC, suggesting that candidate biomarkers may provide complementary information when combined with conventional clinical markers. Detailed AUC values, 95% CIs, sensitivities, specificities, and optimal cutoff values for individual biomarkers are presented in Table 3, and those for combined biomarker models are presented in Table 4, with corresponding ROC curves shown in Figures 7 and 8.

**TABLE 3:** Diagnostic performance of individual biomarkers in the discovery cohort.

Biomarker	AUC	95% CI, lower	95% CI, upper	Optimal cutoff	Sensitivity	Specificity
CA19‐9	0.925	0.877	0.973	21.000	0.869	1.000
MARCO	0.857	0.781	0.932	0.3959	0.788	0.800
PEP‐FAP	0.844	0.760	0.929	0.6603	0.929	0.667
Trypsin‐2	0.806	0.722	0.889	0.3784	0.768	0.800
REG1A	0.784	0.696	0.872	0.7043	0.707	0.867
Cathepsin S (Cath‐S)	0.717	0.607	0.826	0.3585	0.869	0.500
PIGR	0.706	0.614	0.798	1.5723	0.535	0.833
CTRB2	0.638	0.543	0.733	1.0385	0.364	1.000
PRDX6	0.603	0.505	0.701	1.4617	0.374	1.000

*Note:* The discovery cohort included 99 patients with PDAC and 30 healthy controls. Optimal cutoff values were determined using the Youden index. Sensitivity and specificity are reported at the optimal cutoff.

Abbreviations: AUC, area under the receiver operating characteristic curve; CA19‐9, carbohydrate antigen 19‐9; CI, confidence interval.

**TABLE 4:** Diagnostic performance of combined biomarker models in the discovery cohort.

Model	AUC	95% CI, lower	95% CI, upper	Optimal cutoff	Sensitivity	Specificity
CA19‐9 + MARCO + PEP‐FAP	0.989	0.976	1.000	0.7866	0.939	1.000
CA19‐9	0.925	0.877	0.973	21.000	0.869	1.000
MARCO + PEP‐FAP	0.911	0.859	0.963	0.7236	0.849	0.833
MARCO	0.857	0.781	0.932	0.3959	0.788	0.800
PEP‐FAP	0.844	0.760	0.929	0.6603	0.929	0.667

*Note:* Combined models were constructed using binary logistic regression. Optimal cutoff values were determined using the Youden index. Sensitivity and specificity are reported at the optimal cutoff.

Abbreviations: AUC, area under the receiver operating characteristic curve; CA19‐9, carbohydrate antigen 19‐9; CI, confidence interval.

### 3.6 Exploratory Diagnostic Performance in the Independent ELISA Cohort

To provide orthogonal support for the discovery‐phase findings, exploratory ROC analyses were performed in the independent ELISA validation subset comprising 10 patients with PDAC and 10 controls. Despite the small sample size, several biomarkers retained high apparent discriminatory ability, and their directions of change were broadly consistent with those observed in the discovery cohort. The exploratory MARCO + PEP‐FAP model also showed favorable classification performance in this subset.

These results, however, should be interpreted with caution. The ELISA cohort was designed as a pilot orthogonal validation subset, not as a definitive diagnostic validation cohort; therefore, the resulting AUC estimates may be unstable. Accordingly, these analyses are presented as supportive and hypothesis‐generating findings. The corresponding ROC curves are shown in Figures S1 and S2, and detailed diagnostic performance of individual biomarkers and combined models is provided in Tables S3 and S4, respectively. The directionally favorable performance of the MARCO + PEP‐FAP model further supports its potential as a biologically informed multi‐marker strategy.

## 4 Discussion

In this study, we used a DIA‐based plasma proteomic workflow followed by targeted ELISA validation to identify circulating proteins associated with PDAC. Among 565 reliably quantified proteins, 52 were differentially expressed between patients with PDAC and healthy controls. ELISA validation further confirmed significantly elevated plasma levels of eight candidate proteins, including Cath‐S, CTRB2, MARCO, PIGR, PRDX6, REG1A, Trypsin‐2, and PEP‐FAP. These findings suggest that PDAC is accompanied by detectable systemic proteomic alterations and support the potential value of these proteins as exploratory diagnostic biomarker candidates.

The biological relevance of these candidates is consistent with known PDAC‐associated physiological disturbances. Several validated proteins, including CTRB2, Trypsin‐2, Cath‐S, and PEP‐FAP, are related to protease activity, digestive enzyme regulation, and extracellular matrix remodeling [16, 17, 18, 19, 20, 21, 22, 23, 24, 25, 26]. Their altered plasma levels may reflect exocrine dysfunction, acinar injury, aberrant zymogen‐related processes, and stromal remodeling, all of which are closely linked to PDAC biology. Consistently, pancreatic secretion and extracellular matrix–related processes were enriched in the pathway analysis, suggesting that tumor‐associated pancreatic and stromal alterations can be captured in the circulating proteome.

In addition to protease‐related changes, several candidates were associated with immune remodeling, epithelial alteration, and oxidative stress. MARCO, a macrophage‐associated receptor, may reflect myeloid remodeling within the PDAC microenvironment [27]. PIGR may indicate epithelial remodeling and altered vesicle‐associated processes [28, 29], whereas PRDX6 may represent oxidative stress and inflammatory pressure associated with malignancy [30, 31]. REG1A, a marker of acinar cell stress and pancreatic injury, was also significantly elevated, consistent with prior reports in pancreatic cancer and inflammatory pancreatic diseases [32, 33, 34]. Together, these findings indicate that the identified plasma proteins likely reflect multiple dimensions of PDAC‐associated systemic disturbance, including exocrine dysfunction, stromal remodeling, immune activation, and injury‐related responses.

Our findings are also consistent with the broader landscape of PDAC liquid biopsy and proteomic research. Previous studies have repeatedly implicated extracellular matrix remodeling, proteolytic activity, inflammatory signaling, and metabolic dysregulation in PDAC [18, 23]. The enrichment of complement and coagulation cascades, cholesterol metabolism, pancreatic secretion, and extracellular processes in our dataset further supports the biological coherence of the observed proteomic signature [35, 36, 37, 38, 39]. Importantly, several candidates identified here, including CTRB2, PEP‐FAP, and MARCO, remain relatively underexplored as circulating biomarkers, suggesting that they may provide useful components for future multi‐marker diagnostic panels.

To assess the translational relevance of these candidates, we performed ROC analyses in both the discovery cohort and the independent ELISA subset. In the discovery cohort, several markers showed moderate‐to‐good discriminatory performance, with MARCO, Trypsin‐2, REG1A, and PEP‐FAP among the stronger individual candidates, while CA19‐9 showed the highest individual AUC. The combination of MARCO and PEP‐FAP improved discrimination compared with either marker alone, and further improvement was observed after incorporating CA19‐9. This supports the concept that biologically complementary markers may provide nonredundant diagnostic information. However, these analyses remain exploratory, particularly in the small ELISA subset, and should not be interpreted as definitive evidence of clinical diagnostic utility.

Tumor location and obstructive jaundice may influence circulating biomarker profiles and therefore warrant careful consideration [40, 41, 42, 43]. Tumors arising in the pancreatic head or uncinate process are more likely to cause biliary obstruction and related systemic changes, whereas non‐head tumors may follow different clinical and biological patterns [40, 42, 44]. In our exploratory location‐stratified analysis, several biomarkers appeared to differ between head‐dominant and non‐head tumors, suggesting that anatomical heterogeneity may contribute to plasma protein variation. Similarly, jaundice was associated with higher levels of PIGR, Cath‐S, and MARCO, indicating that some biomarker alterations may partly reflect cholestasis‐related or biliary inflammatory effects rather than tumor‐specific biology alone. By contrast, PEP‐FAP, PRDX6, CTRB2, REG1A, and Trypsin‐2 did not differ significantly according to jaundice status. These subgroup findings are preliminary and require confirmation in larger, anatomically balanced and clinically annotated cohorts.

Several limitations should be acknowledged. First, this was a single‐center, cross‐sectional case–control study using healthy individuals as the only control group, which limits assessment of biomarker specificity in clinically relevant differential diagnostic settings. Second, the independent ELISA cohort was small and designed as a pilot orthogonal validation subset rather than a definitive clinical validation cohort; therefore, the effect‐size estimates and diagnostic performance metrics from this subset should be interpreted cautiously. Third, potential confounding by obstructive jaundice and tumor‐location imbalance may have influenced circulating protein profiles and limited the interpretation of subgroup analyses. Larger multicenter studies incorporating clinically relevant disease controls, external validation cohorts, and longitudinal sampling are needed to confirm the robustness and clinical utility of these findings.

Future studies should validate these candidate biomarkers in larger, multicenter cohorts that include clinically relevant disease controls. Longitudinal sampling in high‐risk populations will be needed to determine whether these proteins can support earlier detection before conventional clinical diagnosis. More rigorous diagnostic modeling should also evaluate optimal multi‐marker panels and their incremental value beyond CA19‐9. In parallel, mechanistic studies of selected candidates may help clarify how PDAC‐associated proteomic alterations relate to pancreatic injury, stromal remodeling, immune activation, and disease progression.

## 5 Conclusion

This proteomic study identified eight plasma proteins with significant differential expression in patients with PDAC and revealed pathway‐level alterations related to protease activity, immune remodeling, oxidative stress, and pancreatic secretion. ROC analyses supported the discriminatory potential of several candidate biomarkers, while exploratory combined modeling suggested that multi‐marker strategies may improve classification beyond individual analytes. Although validation in larger and clinically diverse cohorts, especially those including relevant disease control groups, remains necessary, these findings provide a basis for developing blood‐based biomarker panels for PDAC detection.

## Funding

This work was supported by the Department of Science and Technology of Sichuan Province (2024YFHZ0074); Health Commission of Sichuan Province (Sichuan Provincial Cadre Health Care Research Project, grant no. 2026‐122); West China Hospital, Sichuan University (Horizontal Scientific Research Project, grant no. 311190572); and Jiangsu Hengrui Pharmaceuticals Co. Ltd. (grant no. 322250332). The funding sources had no role in the study design, data collection, analysis, interpretation, or manuscript preparation.

## Ethics Statement

The study was conducted in accordance with the Declaration of Helsinki and was approved by the Ethics Committee of West China Hospital of Sichuan University (no. 2022‐597).

## Conflicts of Interest

The authors declare no conflicts of interest.

## Supporting information


**Figure S1:** Exploratory ROC curves of ELISA‐validated biomarkers in the independent validation cohort.
**Figure S2:** Exploratory ROC curves of selected biomarkers and the combined model in the independent validation cohort.
**Table S1:** Associations between clinical variables and candidate biomarker levels within the PDAC cohort.
**Table S2:** Exploratory subgroup analysis according to tumor anatomical location in patients with PDAC.
**Table S3:** Exploratory diagnostic performance of candidate biomarkers in the independent ELISA validation cohort.
**Table S4:** Exploratory diagnostic performance of selected individual biomarkers and combined models in the independent ELISA validation cohort.

## Data Availability

The data that support the findings of this study are available from the corresponding author upon reasonable request.
