# Supplementary material for: Plasma Proteomic Profiling Identifies Candidate Biomarkers for Pancreatic Ductal Adenocarcinoma
Source: J Clin Lab Anal. 2026 Jul 21;40(17):e70313. doi: 10.1002/jcla.70313 (PMC13399626; doi:10.1002/jcla.70313)
Supplement: Supplementary file 1 [file JCLA-40-e70313-s001.docx]

**Plasma Proteomic Profiling Identifies Candidate Biomarkers for Pancreatic Ductal Adenocarcinoma**

**Supplementary Materials**

**Contents**

Figure S1. Exploratory ROC curves of ELISA-validated biomarkers in the independent validation cohort	2

Figure S2. Exploratory ROC curves of selected biomarkers and the combined model in the independent validation cohort	3

Table S1. Associations between clinical variables and candidate biomarker levels within the PDAC cohort	4

Table S2. Exploratory subgroup analysis according to tumor anatomical location in patients with PDAC	5

Table S3. Exploratory diagnostic performance of candidate biomarkers in the independent ELISA validation cohort	6

Table S4. Exploratory diagnostic performance of selected individual biomarkers and combined models in the independent ELISA validation cohort	8

**Figure S1. Exploratory ROC curves of ELISA-validated biomarkers in the independent validation cohort.**

ROC curves were generated in the independent ELISA validation cohort consisting of 10 patients with PDAC and 10 healthy controls. Several biomarkers showed high apparent discriminatory performance, including Trypsin-2 (AUC = 0.970), MARCO (AUC = 0.970), REG1A (AUC = 0.930), CTRB2 (AUC = 0.920), PIGR (AUC = 0.910), PRDX6 (AUC = 0.910), PEP-FAP (AUC = 0.910), and Cathepsin S (Cath-S; AUC = 0.900). The diagonal dashed line indicates no-discrimination performance (AUC = 0.500). Because of the limited sample size, these analyses should be interpreted as exploratory.

**Figure S2. Exploratory ROC curves of selected biomarkers and the combined model in the independent validation cohort.**

MARCO showed strong apparent discriminatory performance (AUC = 0.970), whereas PEP-FAP showed an AUC of 0.910. The combined MARCO + PEP-FAP model yielded the highest apparent performance (AUC = 0.980), exceeding either marker alone. The diagonal dashed line indicates no-discrimination performance (AUC = 0.500). Because of the limited sample size, these analyses should be interpreted as exploratory.

**Table S1. Associations between clinical variables and candidate biomarker levels within the PDAC cohort.**

| **Clinical variable** | **Biomarker** | **No, n** | **Yes, n** | **No group** | **Yes group** | **P value** | **FDR-adjusted P value** |
| --- | --- | --- | --- | --- | --- | --- | --- |
| Jaundice | PIGR | 71 | 28 | 1.206 (0.953, 1.91) | 9.545 (6.77, 12.793) | <0.001 | <0.001 |
| Jaundice | REG1A | 71 | 28 | 0.871 (0.573, 1.622) | 1.085 (0.616, 1.391) | 0.88 | 0.936 |
| Jaundice | Trypsin-2 | 71 | 28 | 0.68 (0.38, 1.307) | 0.567 (0.416, 1.137) | 0.935 | 0.936 |
| Jaundice | Cath-S | 71 | 28 | 0.547 (0.391, 0.946) | 2.888 (0.82, 4.046) | <0.001 | 0.00111 |
| Jaundice | PRDX6 | 71 | 28 | 0.525 (0.365, 6.471) | 0.622 (0.377, 4.846) | 0.783 | 0.936 |
| Jaundice | PEP-FAP | 71 | 28 | 0.373 (0.278, 0.514) | 0.347 (0.256, 0.446) | 0.233 | 0.723 |
| Jaundice | CTRB2 | 71 | 28 | 0.712 (0.486, 2.028) | 0.712 (0.526, 0.988) | 0.855 | 0.936 |
| Jaundice | MARCO | 71 | 28 | 0.548 (0.392, 1.018) | 1.45 (0.64, 2.939) | 0.00177 | 0.026 |
| Jaundice | CA19-9 | 71 | 28 | 158 (35.05, 723.5) | 226 (105.375, 875) | 0.1 | 0.501 |
| Smoking | PIGR | 66 | 33 | 1.338 (0.962, 5.217) | 2.174 (1.517, 9.417) | 0.00442 | 0.05 |
| Smoking | REG1A | 66 | 33 | 0.858 (0.551, 1.371) | 1.067 (0.643, 1.73) | 0.185 | 0.68 |
| Smoking | Trypsin-2 | 66 | 33 | 0.625 (0.384, 1.302) | 0.734 (0.372, 1.159) | 0.885 | 0.936 |
| Smoking | Cath-S | 66 | 33 | 0.701 (0.429, 3.015) | 0.599 (0.399, 1.788) | 0.58 | 0.816 |
| Smoking | PRDX6 | 66 | 33 | 0.544 (0.356, 5.656) | 0.566 (0.375, 5.361) | 0.686 | 0.882 |
| Smoking | PEP-FAP | 66 | 33 | 0.374 (0.27, 0.49) | 0.367 (0.272, 0.48) | 0.575 | 0.816 |
| Smoking | CTRB2 | 66 | 33 | 0.661 (0.487, 1.891) | 0.817 (0.528, 1.429) | 0.404 | 0.757 |
| Smoking | MARCO | 66 | 33 | 0.643 (0.454, 1.398) | 0.882 (0.426, 1.912) | 0.506 | 0.814 |
| Smoking | CA19-9 | 66 | 33 | 177 (56.25, 931) | 165 (33, 450) | 0.388 | 0.757 |
| Hypertension | PIGR | 77 | 22 | 1.658 (1.116, 6.52) | 1.612 (0.999, 4.579) | 0.547 | 0.816 |
| Hypertension | REG1A | 77 | 22 | 0.888 (0.588,1.453) | 1.018 (0.58, 1.286) | 0.936 | 0.936 |
| Hypertension | Trypsin-2 | 77 | 22 | 0.653 (0.381, 1.309) | 0.482 (0.381, 1.064) | 0.331 | 0.745 |
| Hypertension | Cath-S | 77 | 22 | 0.746 (0.442, 3.216) | 0.436 (0.36, 0.697) | 0.017 | 0.15 |
| Hypertension | PRDX6 | 77 | 22 | 0.586 (0.379, 6.401) | 0.427 (0.338, 2.717) | 0.196 | 0.68 |
| Hypertension | PEP-FAP | 77 | 22 | 0.367 (0.262, 0.494) | 0.396 (0.315, 0.477) | 0.531 | 0.816 |
| Hypertension | CTRB2 | 77 | 22 | 0.791 (0.497, 2.123) | 0.529 (0.456, 0.902) | 0.067 | 0.378 |
| Hypertension | MARCO | 77 | 22 | 0.629 (0.426, 1.606) | 0.682 (0.567, 3.052) | 0.169 | 0.68 |
| Hypertension | CA19-9 | 77 | 22 | 158 (40, 688) | 226 (54.7, 948.75) | 0.297 | 0.723 |
| Diabetes | PIGR | 80 | 19 | 1.612 (1.066, 5.701) | 1.907 (1.199, 7.222) | 0.276 | 0.723 |
| Diabetes | REG1A | 80 | 19 | 0.879 (0.554, 1.419) | 1.201 (0.718, 1.691) | 0.442 | 0.796 |
| Diabetes | Trypsin-2 | 80 | 19 | 0.692 (0.381, 1.353) | 0.518 (0.382, 0.895) | 0.139 | 0.625 |
| Diabetes | Cath-S | 80 | 19 | 0.689 (0.419, 2.576) | 0.529 (0.37, 3.473) | 0.647 | 0.857 |
| Diabetes | PRDX6 | 80 | 19 | 0.531 (0.361, 5.199) | 0.646 (0.37, 5.651) | 0.502 | 0.814 |
| Diabetes | PEP-FAP | 80 | 19 | 0.368 (0.271, 0.479) | 0.374 (0.273, 0.535) | 0.842 | 0.936 |
| Diabetes | CTRB2 | 80 | 19 | 0.804 (0.502, 2.256) | 0.533 (0.442, 0.771) | 0.028 | 0.179 |
| Diabetes | MARCO | 80 | 19 | 0.635 (0.435, 1.476) | 0.883 (0.468, 2.979) | 0.288 | 0.723 |
| Diabetes | CA19-9 | 80 | 19 | 148.5 (36.025, 705.75) | 440 (193, 923.5) | 0.028 | 0.179 |
| Drinking | PIGR | 72 | 27 | 1.51 (1.066, 6.277) | 1.914 (1.464, 5.137) | 0.305 | 0.723 |
| Drinking | REG1A | 72 | 27 | 0.988 (0.686, 1.469) | 0.807 (0.563, 1.347) | 0.368 | 0.757 |
| Drinking | Trypsin-2 | 72 | 27 | 0.66 (0.388, 1.295) | 0.556 (0.366, 1.11) | 0.377 | 0.757 |
| Drinking | Cath-S | 72 | 27 | 0.684 (0.429, 3.1) | 0.599 (0.382, 1.229) | 0.283 | 0.723 |
| Drinking | PRDX6 | 72 | 27 | 0.553 (0.363, 4.991) | 0.566 (0.384, 6.639) | 0.607 | 0.827 |
| Drinking | PEP-FAP | 72 | 27 | 0.358 (0.271, 0.477) | 0.417 (0.268, 0.508) | 0.811 | 0.936 |
| Drinking | CTRB2 | 72 | 27 | 0.682 (0.489, 1.981) | 0.791 (0.5, 1.091) | 0.928 | 0.936 |
| Drinking | MARCO | 72 | 27 | 0.664 (0.469, 1.705) | 0.523 (0.349, 1.855) | 0.463 | 0.801 |
| Drinking | CA19-9 | 72 | 27 | 175 (38.35, 781.5) | 178 (49.75, 626) | 0.847 | 0.936 |

Data are presented as median (IQR). Group comparisons were performed using the Mann-Whitney U test. FDR-adjusted P values were calculated using the Benjamini-Hochberg method. The “No group” and “Yes group” columns refer to the absence or presence of the corresponding clinical variable. PDAC, pancreatic ductal adenocarcinoma; IQR, interquartile range; FDR, false discovery rate; CA19-9, carbohydrate antigen 19-9; PIGR, polymeric immunoglobulin receptor; REG1A, lithostathine-1-alpha; Cath-S, Cathepsin S; PRDX6, peroxiredoxin-6; PEP-FAP, prolyl endopeptidase fibrinolytic activity protein; CTRB2, chymotrypsinogen B2; MARCO, macrophage receptor with collagenous structure.

**Table S2. Exploratory subgroup analysis according to tumor anatomical location in patients with PDAC**

| **Variable** | **Head/uncinate (n=56)** | **Body/tail (n=43)** | **P value** | **Statistical test** |
| --- | --- | --- | --- | --- |
| Hypertension, n (%) | 9/56 (16.1) | 13/43 (30.2) | 0.142 | Fisher exact test |
| Diabetes mellitus, n (%) | 10/56 (17.9) | 9/43 (20.9) | 0.799 | Fisher exact test |
| Smoking history, n (%) | 17/56 (30.4) | 16/43 (37.2) | 0.523 | Fisher exact test |
| Drinking history, n (%) | 12/56 (21.4) | 15/43 (34.9) | 0.173 | Fisher exact test |
| **Jaundice, n (%)** | 26/56 (46.4) | 2/43 (4.7) | **<0.001** | Fisher exact test |
| CA19-9, U/mL | 176.000 (59.175–564.000) | 176.000 (27.000–897.500) | 0.854 | Mann–Whitney U test |
| **PIGR** | 3.623 (1.164–9.528) | 1.393 (0.969–1.849) | **<0.001** | Mann–Whitney U test |
| REG1A | 1.116 (0.643–1.780) | 0.786 (0.554–1.168) | 0.067 | Mann–Whitney U test |
| Trypsin-2 | 0.575 (0.403–1.295) | 0.680 (0.366–1.170) | 0.518 | Mann–Whitney U test |
| **Cath-S** | 0.824 (0.501–3.307) | 0.486 (0.389–0.946) | **0.012** | Mann–Whitney U test |
| PRDX6 | 0.597 (0.344–6.092) | 0.485 (0.374–4.616) | 0.764 | Mann–Whitney U test |
| PEP-FAP | 0.355 (0.265–0.437) | 0.426 (0.289–0.546) | 0.062 | Mann–Whitney U test |
| **CTRB2** | 0.942 (0.580–2.397) | 0.518 (0.443–0.863) | **<0.001** | Mann–Whitney U test |
| MARCO | 0.728 (0.462–1.978) | 0.557 (0.369–1.035) | 0.181 | Mann–Whitney U test |

Abbreviations: PDAC, pancreatic ductal adenocarcinoma; IQR, interquartile range; CA19-9, carbohydrate antigen 19-9. Data are presented as n/N (%) for categorical variables and median (IQR) for continuous variables. Location was coded as 0 for pancreatic head/uncinate and 1 for pancreatic body/tail. P values were calculated using Fisher exact test for categorical variables and Mann–Whitney U test for continuous variables. Bold P values indicate statistical significance (P<0.05).

**Table S3. Exploratory diagnostic performance of candidate biomarkers in the independent ELISA validation cohort.**

| **Biomarker** | **AUC** | **95% CI, lower** | **95% CI, upper** | **Optimal cut-off** | **Sensitivity** | **Specificity** |
| --- | --- | --- | --- | --- | --- | --- |
| Trypsin-2 | 0.97 | 0.904 | 1.000 | 42.044 | 0.9 | 1.000 |
| MARCO | 0.97 | 0.911 | 1.000 | 0.949 | 1.000 | 0.8 |
| REG1A | 0.93 | 0.817 | 1.000 | 91.987 | 0.9 | 0.9 |
| CTRB2 | 0.92 | 0.805 | 1.000 | 4.831 | 0.9 | 0.8 |
| PIGR | 0.91 | 0.776 | 1.000 | 3.224 | 0.8 | 1.000 |
| PRDX6 | 0.91 | 0.764 | 1.000 | 1.603 | 0.9 | 0.9 |
| PEP-FAP | 0.91 | 0.786 | 1.000 | 1169.515 | 1.000 | 0.7 |
| Cathepsin S (Cath-S) | 0.9 | 0.763 | 1.000 | 83.801 | 0.9 | 0.8 |

ROC, receiver operating characteristic; AUC, area under the curve; CI, confidence interval; ELISA, enzyme-linked immunosorbent assay; PDAC, pancreatic ductal adenocarcinoma. The independent validation cohort included 10 patients with PDAC and 10 healthy controls. Optimal cut-off values were determined using the Youden index. Sensitivity and specificity are reported at the optimal cut-off. These analyses were exploratory because of the limited sample size of the validation cohort.

**Table S4. Exploratory diagnostic performance of selected individual biomarkers and combined models in the independent ELISA validation cohort.**

| **Model** | **AUC** | **95% CI, lower** | **95% CI, upper** | **Optimal cut-off** | **Sensitivity** | **Specificity** |
| --- | --- | --- | --- | --- | --- | --- |
| MARCO | 0.97 | 0.911 | 1.000 | 0.949 | 1.000 | 0.8 |
| PEP-FAP | 0.91 | 0.786 | 1.000 | 1169.514 | 1.000 | 0.7 |
| MARCO + PEP-FAP | 0.98 | 0.933 | 1.000 | 0.702 | 0.9 | 1.000 |

ROC, receiver operating characteristic; AUC, area under the curve; CI, confidence interval; ELISA, enzyme-linked immunosorbent assay; PDAC, pancreatic ductal adenocarcinoma. The independent validation cohort included 10 patients with PDAC and 10 healthy controls. Combined models were constructed using binary logistic regression. Optimal cut-off values were determined using the Youden index. Sensitivity and specificity are reported at the optimal cut-off. These analyses were exploratory because of the limited sample size.
