# Supplementary material for: Plasma Proteomic Profiling Identifies Candidate Biomarkers for Pancreatic Ductal Adenocarcinoma
Source: J Clin Lab Anal. 2026 Jul 21:e70313. Online ahead of print. doi: 10.1002/jcla.70313 (PMC13399626; doi:10.1002/jcla.70313)
Supplement: Supplementary file 1 — Figure S1: Exploratory ROC curves of ELISA‐validated biomarkers in the independent validation cohort. Figure S2: Exploratory ROC curves of selected biomarkers and the combined model in the independent validation cohort. Table S1: Associations between clinical variables and candidate biomarker levels within the PDAC cohort. Table S2: Exploratory subgroup analysis according to tumor anatomical location in patients with PDAC. Table S3: Exploratory diagnostic performance of candidate biomarkers in the independent ELISA validation cohort. Table S4: Exploratory diagnostic performance of selected individual biomarkers and combined models in the independent ELISA validation cohort. [file JCLA-9999-e70313-s001.docx]

**Plasma Proteomic Profiling Identifies Candidate Biomarkers for Pancreatic Ductal Adenocarcinoma**

**Supplementary Materials**

**Contents**

Figure S1. Exploratory ROC curves of ELISA-validated biomarkers in the independent validation cohort2

Figure S2. Exploratory ROC curves of selected biomarkers and the combined model in the independent validation cohort3

Table S1. Associations between clinical variables and candidate biomarker levels within the PDAC cohort4

Table S2. Exploratory subgroup analysis according to tumor anatomical location in patients with PDAC 5

Table S3. Exploratory diagnostic performance of candidate biomarkers in the independent ELISA validation cohort 6

Table S4. Exploratory diagnostic performance of selected individual biomarkers and combined models in the independent ELISA validation cohort 8

**Figure S1. Exploratory ROC curves of ELISA-validated biomarkers in the independent validation cohort.**


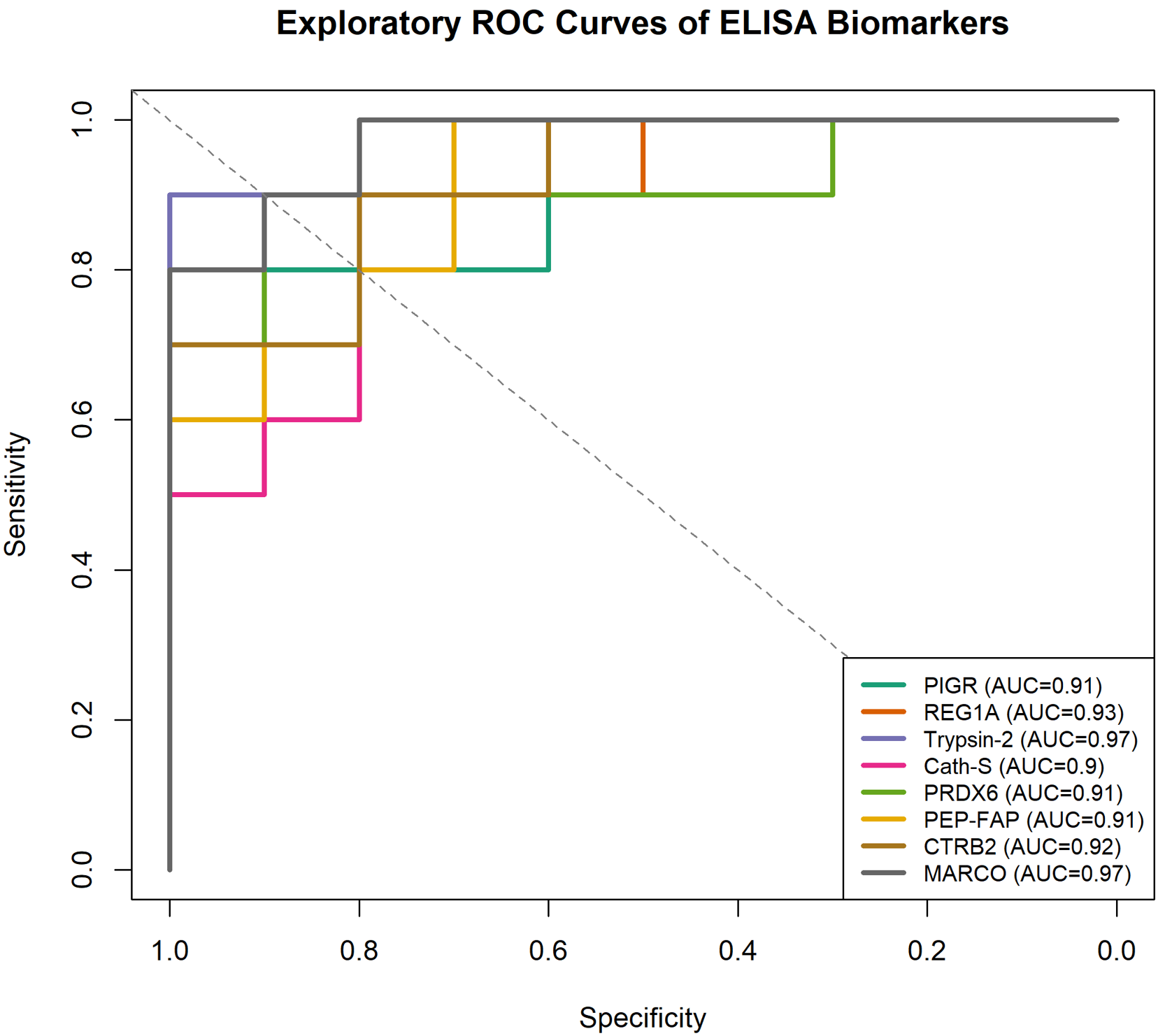


ROC curves were generated in the independent ELISA validation cohort consisting of 10 patients with PDAC and 10 healthy controls. Several biomarkers showed high apparent discriminatory performance, including Trypsin-2 (AUC = 0.970), MARCO (AUC = 0.970), REG1A (AUC = 0.930), CTRB2 (AUC = 0.920), PIGR (AUC = 0.910), PRDX6 (AUC = 0.910), PEP-FAP (AUC = 0.910), and Cathepsin S (Cath-S; AUC = 0.900). The diagonal dashed line indicates no-discrimination performance (AUC = 0.500). Because of the limited sample size, these analyses should be interpreted as exploratory.

**Figure S2. Exploratory ROC curves of selected biomarkers and the combined model in the independent validation cohort.**


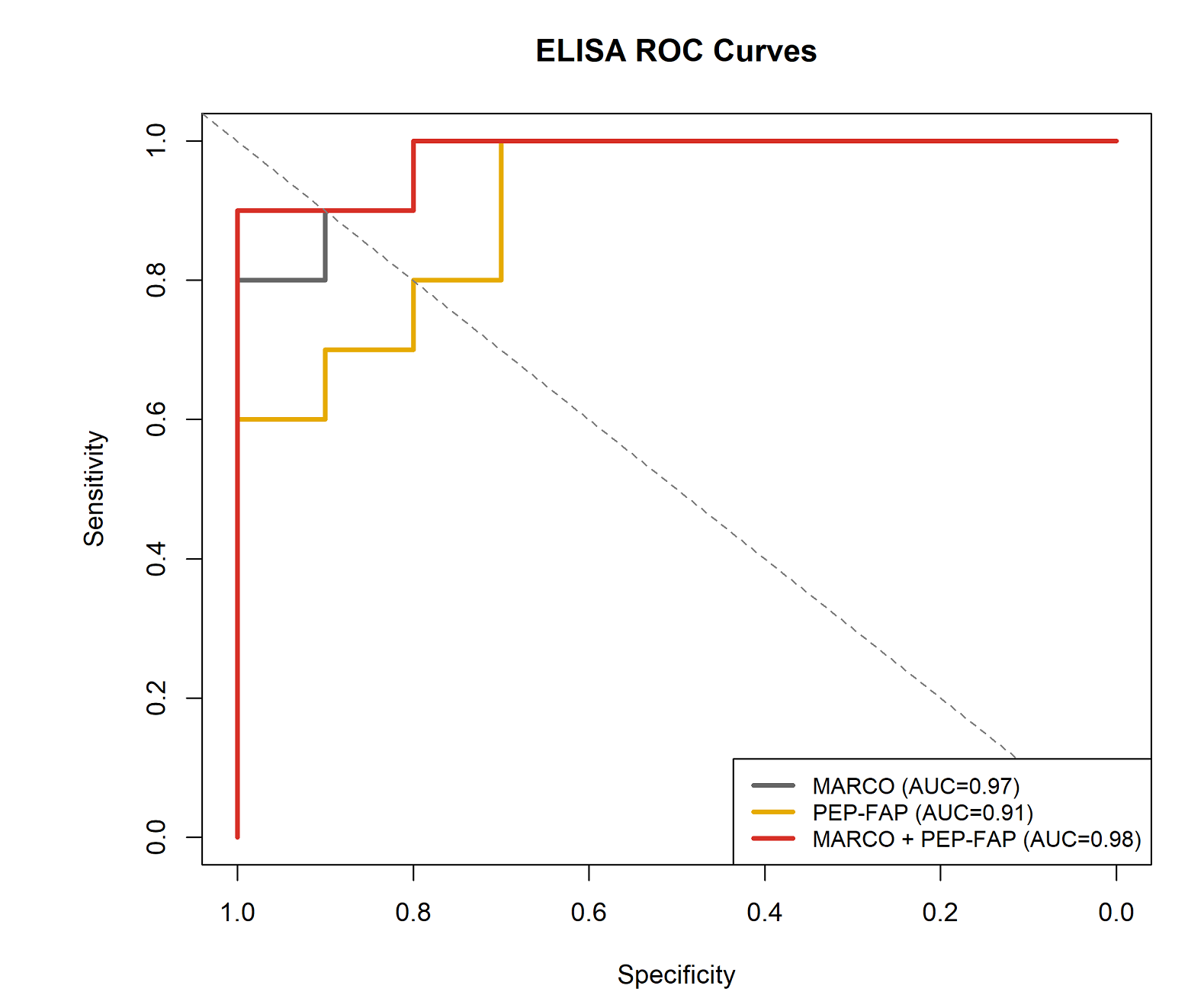


MARCO showed strong apparent discriminatory performance (AUC = 0.970), whereas PEP-FAP showed an AUC of 0.910. The combined MARCO + PEP-FAP model yielded the highest apparent performance (AUC = 0.980), exceeding either marker alone. The diagonal dashed line indicates no-discrimination performance (AUC = 0.500). Because of the limited sample size, these analyses should be interpreted as exploratory.

**Table S1. Associations between clinical variables and candidate biomarker levels within the PDAC cohort.**

| **Clinical variable** | **Biomarker** | **No, n** | **Yes, n** | **No group** | **Yes group** | **P value** | **FDR-adjusted P value** |
| --- | --- | --- | --- | --- | --- | --- | --- |
| Jaundice | PIGR | 71 | 28 | 1.206 (0.953, 1.91) | 9.545 (6.77, 12.793) | <0.001 | <0.001 |
| Jaundice | REG1A | 71 | 28 | 0.871 (0.573, 1.622) | 1.085 (0.616, 1.391) | 0.88 | 0.936 |
| Jaundice | Trypsin-2 | 71 | 28 | 0.68 (0.38, 1.307) | 0.567 (0.416, 1.137) | 0.935 | 0.936 |
| Jaundice | Cath-S | 71 | 28 | 0.547 (0.391, 0.946) | 2.888 (0.82, 4.046) | <0.001 | 0.00111 |
| Jaundice | PRDX6 | 71 | 28 | 0.525 (0.365, 6.471) | 0.622 (0.377, 4.846) | 0.783 | 0.936 |
| Jaundice | PEP-FAP | 71 | 28 | 0.373 (0.278, 0.514) | 0.347 (0.256, 0.446) | 0.233 | 0.723 |
| Jaundice | CTRB2 | 71 | 28 | 0.712 (0.486, 2.028) | 0.712 (0.526, 0.988) | 0.855 | 0.936 |
| Jaundice | MARCO | 71 | 28 | 0.548 (0.392, 1.018) | 1.45 (0.64, 2.939) | 0.00177 | 0.026 |
| Jaundice | CA19-9 | 71 | 28 | 158 (35.05, 723.5) | 226 (105.375, 875) | 0.1 | 0.501 |
| Smoking | PIGR | 66 | 33 | 1.338 (0.962, 5.217) | 2.174 (1.517, 9.417) | 0.00442 | 0.05 |
| Smoking | REG1A | 66 | 33 | 0.858 (0.551, 1.371) | 1.067 (0.643, 1.73) | 0.185 | 0.68 |
| Smoking | Trypsin-2 | 66 | 33 | 0.625 (0.384, 1.302) | 0.734 (0.372, 1.159) | 0.885 | 0.936 |
| Smoking | Cath-S | 66 | 33 | 0.701 (0.429, 3.015) | 0.599 (0.399, 1.788) | 0.58 | 0.816 |
| Smoking | PRDX6 | 66 | 33 | 0.544 (0.356, 5.656) | 0.566 (0.375, 5.361) | 0.686 | 0.882 |
| Smoking | PEP-FAP | 66 | 33 | 0.374 (0.27, 0.49) | 0.367 (0.272, 0.48) | 0.575 | 0.816 |
| Smoking | CTRB2 | 66 | 33 | 0.661 (0.487, 1.891) | 0.817 (0.528, 1.429) | 0.404 | 0.757 |
| Smoking | MARCO | 66 | 33 | 0.643 (0.454, 1.398) | 0.882 (0.426, 1.912) | 0.506 | 0.814 |
| Smoking | CA19-9 | 66 | 33 | 177 (56.25, 931) | 165 (33, 450) | 0.388 | 0.757 |
| Hypertension | PIGR | 77 | 22 | 1.658 (1.116, 6.52) | 1.612 (0.999, 4.579) | 0.547 | 0.816 |
| Hypertension | REG1A | 77 | 22 | 0.888 (0.588,1.453) | 1.018 (0.58, 1.286) | 0.936 | 0.936 |
| Hypertension | Trypsin-2 | 77 | 22 | 0.653 (0.381, 1.309) | 0.482 (0.381, 1.064) | 0.331 | 0.745 |
| Hypertension | Cath-S | 77 | 22 | 0.746 (0.442, 3.216) | 0.436 (0.36, 0.697) | 0.017 | 0.15 |
| Hypertension | PRDX6 | 77 | 22 | 0.586 (0.379, 6.401) | 0.427 (0.338, 2.717) | 0.196 | 0.68 |
| Hypertension | PEP-FAP | 77 | 22 | 0.367 (0.262, 0.494) | 0.396 (0.315, 0.477) | 0.531 | 0.816 |
| Hypertension | CTRB2 | 77 | 22 | 0.791 (0.497, 2.123) | 0.529 (0.456, 0.902) | 0.067 | 0.378 |
| Hypertension | MARCO | 77 | 22 | 0.629 (0.426, 1.606) | 0.682 (0.567, 3.052) | 0.169 | 0.68 |
| Hypertension | CA19-9 | 77 | 22 | 158 (40, 688) | 226 (54.7, 948.75) | 0.297 | 0.723 |
| Diabetes | PIGR | 80 | 19 | 1.612 (1.066, 5.701) | 1.907 (1.199, 7.222) | 0.276 | 0.723 |
| Diabetes | REG1A | 80 | 19 | 0.879 (0.554, 1.419) | 1.201 (0.718, 1.691) | 0.442 | 0.796 |
| Diabetes | Trypsin-2 | 80 | 19 | 0.692 (0.381, 1.353) | 0.518 (0.382, 0.895) | 0.139 | 0.625 |
| Diabetes | Cath-S | 80 | 19 | 0.689 (0.419, 2.576) | 0.529 (0.37, 3.473) | 0.647 | 0.857 |
| Diabetes | PRDX6 | 80 | 19 | 0.531 (0.361, 5.199) | 0.646 (0.37, 5.651) | 0.502 | 0.814 |
| Diabetes | PEP-FAP | 80 | 19 | 0.368 (0.271, 0.479) | 0.374 (0.273, 0.535) | 0.842 | 0.936 |
| Diabetes | CTRB2 | 80 | 19 | 0.804 (0.502, 2.256) | 0.533 (0.442, 0.771) | 0.028 | 0.179 |
| Diabetes | MARCO | 80 | 19 | 0.635 (0.435, 1.476) | 0.883 (0.468, 2.979) | 0.288 | 0.723 |
| Diabetes | CA19-9 | 80 | 19 | 148.5 (36.025, 705.75) | 440 (193, 923.5) | 0.028 | 0.179 |
| Drinking | PIGR | 72 | 27 | 1.51 (1.066, 6.277) | 1.914 (1.464, 5.137) | 0.305 | 0.723 |
| Drinking | REG1A | 72 | 27 | 0.988 (0.686, 1.469) | 0.807 (0.563, 1.347) | 0.368 | 0.757 |
| Drinking | Trypsin-2 | 72 | 27 | 0.66 (0.388, 1.295) | 0.556 (0.366, 1.11) | 0.377 | 0.757 |
| Drinking | Cath-S | 72 | 27 | 0.684 (0.429, 3.1) | 0.599 (0.382, 1.229) | 0.283 | 0.723 |
| Drinking | PRDX6 | 72 | 27 | 0.553 (0.363, 4.991) | 0.566 (0.384, 6.639) | 0.607 | 0.827 |
| Drinking | PEP-FAP | 72 | 27 | 0.358 (0.271, 0.477) | 0.417 (0.268, 0.508) | 0.811 | 0.936 |
| Drinking | CTRB2 | 72 | 27 | 0.682 (0.489, 1.981) | 0.791 (0.5, 1.091) | 0.928 | 0.936 |
| Drinking | MARCO | 72 | 27 | 0.664 (0.469, 1.705) | 0.523 (0.349, 1.855) | 0.463 | 0.801 |
| Drinking | CA19-9 | 72 | 27 | 175 (38.35, 781.5) | 178 (49.75, 626) | 0.847 | 0.936 |

Data are presented as median (IQR). Group comparisons were performed using the Mann-Whitney U test. FDR-adjusted P values were calculated using the Benjamini-Hochberg method. The “No group” and “Yes group” columns refer to the absence or presence of the corresponding clinical variable. PDAC, pancreatic ductal adenocarcinoma; IQR, interquartile range; FDR, false discovery rate; CA19-9, carbohydrate antigen 19-9; PIGR, polymeric immunoglobulin receptor; REG1A, lithostathine-1-alpha; Cath-S, Cathepsin S; PRDX6, peroxiredoxin-6; PEP-FAP, prolyl endopeptidase fibrinolytic activity protein; CTRB2, chymotrypsinogen B2; MARCO, macrophage receptor with collagenous structure.

**Table S2. Exploratory subgroup analysis according to tumor anatomical location in patients with PDAC**

| **Variable** | **Head/uncinate (n=56)** | **Body/tail (n=43)** | **P value** | **Statistical test** |
| --- | --- | --- | --- | --- |
| Hypertension, n (%) | 9/56 (16.1) | 13/43 (30.2) | 0.142 | Fisher exact test |
| Diabetes mellitus, n (%) | 10/56 (17.9) | 9/43 (20.9) | 0.799 | Fisher exact test |
| Smoking history, n (%) | 17/56 (30.4) | 16/43 (37.2) | 0.523 | Fisher exact test |
| Drinking history, n (%) | 12/56 (21.4) | 15/43 (34.9) | 0.173 | Fisher exact test |
| **Jaundice, n (%)** | 26/56 (46.4) | 2/43 (4.7) | **<0.001** | Fisher exact test |
| CA19-9, U/mL | 176.000 (59.175–564.000) | 176.000 (27.000–897.500) | 0.854 | Mann–Whitney U test |
| **PIGR** | 3.623 (1.164–9.528) | 1.393 (0.969–1.849) | **<0.001** | Mann–Whitney U test |
| REG1A | 1.116 (0.643–1.780) | 0.786 (0.554–1.168) | 0.067 | Mann–Whitney U test |
| Trypsin-2 | 0.575 (0.403–1.295) | 0.680 (0.366–1.170) | 0.518 | Mann–Whitney U test |
| **Cath-S** | 0.824 (0.501–3.307) | 0.486 (0.389–0.946) | **0.012** | Mann–Whitney U test |
| PRDX6 | 0.597 (0.344–6.092) | 0.485 (0.374–4.616) | 0.764 | Mann–Whitney U test |
| PEP-FAP | 0.355 (0.265–0.437) | 0.426 (0.289–0.546) | 0.062 | Mann–Whitney U test |
| **CTRB2** | 0.942 (0.580–2.397) | 0.518 (0.443–0.863) | **<0.001** | Mann–Whitney U test |
| MARCO | 0.728 (0.462–1.978) | 0.557 (0.369–1.035) | 0.181 | Mann–Whitney U test |

Abbreviations: PDAC, pancreatic ductal adenocarcinoma; IQR, interquartile range; CA19-9, carbohydrate antigen 19-9. Data are presented as n/N (%) for categorical variables and median (IQR) for continuous variables. Location was coded as 0 for pancreatic head/uncinate and 1 for pancreatic body/tail. P values were calculated using Fisher exact test for categorical variables and Mann–Whitney U test for continuous variables. Bold P values indicate statistical significance (P<0.05).

**Table S3. Exploratory diagnostic performance of candidate biomarkers in the independent ELISA validation cohort.**

| **Biomarker** | **AUC** | **95% CI, lower** | **95% CI, upper** | **Optimal cut-off** | **Sensitivity** | **Specificity** |
| --- | --- | --- | --- | --- | --- | --- |
| Trypsin-2 | 0.97 | 0.904 | 1.000 | 42.044 | 0.9 | 1.000 |
| MARCO | 0.97 | 0.911 | 1.000 | 0.949 | 1.000 | 0.8 |
| REG1A | 0.93 | 0.817 | 1.000 | 91.987 | 0.9 | 0.9 |
| CTRB2 | 0.92 | 0.805 | 1.000 | 4.831 | 0.9 | 0.8 |
| PIGR | 0.91 | 0.776 | 1.000 | 3.224 | 0.8 | 1.000 |
| PRDX6 | 0.91 | 0.764 | 1.000 | 1.603 | 0.9 | 0.9 |
| PEP-FAP | 0.91 | 0.786 | 1.000 | 1169.515 | 1.000 | 0.7 |
| Cathepsin S (Cath-S) | 0.9 | 0.763 | 1.000 | 83.801 | 0.9 | 0.8 |

ROC, receiver operating characteristic; AUC, area under the curve; CI, confidence interval; ELISA, enzyme-linked immunosorbent assay; PDAC, pancreatic ductal adenocarcinoma. The independent validation cohort included 10 patients with PDAC and 10 healthy controls. Optimal cut-off values were determined using the Youden index. Sensitivity and specificity are reported at the optimal cut-off. These analyses were exploratory because of the limited sample size of the validation cohort.

**Table S4. Exploratory diagnostic performance of selected individual biomarkers and combined models in the independent ELISA validation cohort.**

| **Model** | **AUC** | **95% CI, lower** | **95% CI, upper** | **Optimal cut-off** | **Sensitivity** | **Specificity** |
| --- | --- | --- | --- | --- | --- | --- |
| MARCO | 0.97 | 0.911 | 1.000 | 0.949 | 1.000 | 0.8 |
| PEP-FAP | 0.91 | 0.786 | 1.000 | 1169.514 | 1.000 | 0.7 |
| MARCO + PEP-FAP | 0.98 | 0.933 | 1.000 | 0.702 | 0.9 | 1.000 |

ROC, receiver operating characteristic; AUC, area under the curve; CI, confidence interval; ELISA, enzyme-linked immunosorbent assay; PDAC, pancreatic ductal adenocarcinoma. The independent validation cohort included 10 patients with PDAC and 10 healthy controls. Combined models were constructed using binary logistic regression. Optimal cut-off values were determined using the Youden index. Sensitivity and specificity are reported at the optimal cut-off. These analyses were exploratory because of the limited sample size.
